# Nuclear and cytoplasmic LIMK1 enhances human breast cancer progression

**DOI:** 10.1186/1476-4598-10-75

**Published:** 2011-06-18

**Authors:** Brice V McConnell, Karen Koto, Arthur Gutierrez-Hartmann

**Affiliations:** 1 Molecular Biology Program, University of Colorado Denver, 12801 East 17th Ave Aurora, CO 80045, USA; 2 College of Osteopathic Medicine, Touro University, 1310 Johnson Lane, Vallejo, CA 94592, USA; 3 Molecular Biology Program, Departments of Medicine, and of Biochemistry and Molecular Genetics, University of Colorado Denver, 12801 East 17th Ave Aurora, CO 80045, USA

## Abstract

**Background:**

LIM kinase 1 (LIMK1) is expressed in both cytoplasmic and nuclear compartments, and is a key regulator of cytoskeletal organization involved in cell migration and proliferation. LIMK1 levels are increased in several human cancers, with LIMK1 over-expression in prostate and breast cancer cells leading to tumor progression. While it has been presumed that the mechanism by which LIMK1 promotes cancer progression is via its cytoplasmic effects, the role of nuclear vs cytoplasmic LIMK1 in the tumorigenic process has not been examined.

**Results:**

To determine if cytoplasmic or nuclear LIMK1 expression correlated with breast cancer, we performed immunohistochemical (IHC) analysis of breast tissue microarrays (TMAs), The IHC analysis of breast TMAs revealed that 76% of malignant breast tissue samples strongly expressed LIMK1 in the cytoplasm, with 52% of these specimens also expressing nuclear LIMK1. Only 48% of benign breast samples displayed strong cytoplasmic LIMK1 expression and 27% of these expressed nuclear LIMK1. To investigate the respective roles of cytoplamsic and nuclear LIMK1 in breast cancer progression, we targeted GFP-LIMK1 to cytoplasmic and nuclear subcellular compartments by fusing nuclear export signals (NESs) or nuclear localization sequences (NLS), respectively, to the amino-terminus of GFP-LIMK1. Stable pools of MDA-MB-231 cells were generated by retroviral transduction, and fluorescence microscopy revealed that GFP alone (control) and GFP-LIMK1 were each expressed in both the cytoplasm and nucleus of MDA-MB-231 cells, whereas NLS-GFP-LIMK1 was expressed in the nucleus and NES-GFP-LIMK1 was expressed in the cytoplasm. Western blot analyses revealed equal expression of GFP-LIMK1 and NES-GFP-LIMK1, with NLS-GFP-LIMK1 expression being less but equal to endogenous LIMK1. Also, Western blotting revealed increased levels of phospho-cofilin, phospho-FAK, phospho-paxillin, phospho-Src, phospho-AKT, and phospho-Erk1/2 in cells expressing all GFP-LIMK1 fusions, compared to GFP alone. Invasion assays revealed that all GFP-LIMK1 fusions increased MDA-MB-231 cell invasion ~1.5-fold, compared to GFP-only control cells. Tumor xenograft studies in nude mice revealed that MDA-MB-231 cells stably expressing GFP-LIMK, NLS-GFP-LIMK1 and NES-GFP-LIMK1 enhanced tumor growth 2.5-, 1.6- and 4.7-fold, respectively, compared to GFP-alone.

**Conclusion:**

Taken together, these data demonstrate that LIMK1 activity in both the cytoplasmic and nuclear compartments promotes breast cancer progression, underscoring that nuclear LIMK1 contributes to the transforming function of LIMK1.

## Background

The Ser/Thr kinase LIMK1, originally characterized within the central nervous system, serves to regulate actin cytoskeletal dynamics by phosphorylation and inactivation of cofilin [1,2]. The kinase domain of LIMK1 is activated via phosphorylation at Thr 508 [3]. This phosphorylation can be mediated by myotonic dystrophy kinase-related Cdc42-binding kinase alpha (MRCKα), Rho-associated coiled-coil domain kinase (ROCK) and p21-activated kinase (PAK), acting downstream of Rho/Rac/Cdc42 signaling [3-6]. The substrates for LIMK1 are members of the actin depolymerizing factor (ADF) and cofilin family, commonly referred to collectively as cofilin. Serine-3 phosphorylation of cofilin by LIMK1 results in inactivation of cofilin and a subsequent stabilization of actin filaments in the region of LIM kinase activity [1,7]. Opposing the activity of LIM kinase on coflilin are a family of phosphatases, slingshot (SSH) and chronophin (CIN) [8,9]. Activated LIMK1 contributes to formation of key actin structures, such as membrane protrusions, stress fibers, and the contractile ring that forms during cytokinesis [3,6,10,11]. Further, the dynamic assembly and disassembly of actin in membrane structures, such as lamellipodia and filopodia, regulated in part by LIMK1, has been postulated to be the basis for LIMK1-mediated cell motility, and an integral component of LIMK1-mediated cell invasion [12-14].

Structurally, the LIMK1 protein is composed of two N-terminal LIM domains, a central PDZ domain, and a C-terminal kinase domain [15]. Within the PDZ domain, there are two functional nuclear export signals (NES), as well as one nuclear localization signal (NLS) within the kinase domain [15]. The leucine-rich NES sequences are sensitive to inhibition by leptomycin B, as addition of leptomycin B results in nuclear accumulation of LIMK1 within cells that otherwise express predominantly cytoplamic LIMK1. Predictably, deletion of the NLS from the kinase domain abrogates this effect [15]. Immunohistochemical studies in cultured mammalian cell lines, as well as paraformaldehyde (PFA)-fixed mammalian tissues, indicate that the subcellular compartmentalization of LIMK1 within cells is generally cytoplasmic, although many cell types express moderate to strong nuclear LIMK1, in addition to the cytoplasmic component [16]. Although it is clear that LIMK1 protein is expressed in both the cytoplasm and nucleus, the majority of LIMK1 studies have focused on the role of LIMK1 in regulating actin dynamics within the cytoplasm.

Functional studies have found that increasing LIMK1 expression in human breast cancer cell lines results in increased cellular invasion and xenograft tumor growth [13,17]. For example, over-expression of LIMK1 in MDA-MB-231, MCF-7 and MDA-MB-435 human breast cancer cell lines resulted in increased cellular migration and invasion through Matrigel [13,17]. In contrast, inhibiting LIMK1 expression, or blocking LIMK1 activity, reduced the aggressive behavior of human MDA-MB-231 and MDA-MB-435 breast cancer cell lines [13,17]. For example, expression of dominant-negative LIMK1 in breast cancer cell lines resulted in suppression of matrigel invasion *in vitro*, and inhibition of liver, lung and bone metastasis *in vivo *[13,17]. Pharmacological inhibition of upstream regulators of LIMK1, co-expression of nischarin (a protein that specifically binds to and inhibits LIMK1), or RNAi-mediated knockdown of LIMK1, all block LIMK1-mediated cellular invasion [18]. Finally, tumor xenograft assays in female nude mice injected with MDA-MB-435 cells over-expressing LIMK1 resulted in tumors that were larger, more vascularized, and more likely to metastasize to the liver and lungs, compared to controls [17].

Despite these advances in our understanding of LIMK1 functions, several key questions remain regarding the ability of LIMK1 to promote cancer progression. In this regard, one of the most intriguing questions is whether the subcellular localization, cytoplasmic versus nuclear, of LIMK1 affects its ability to promote the transformed phenotype. For example, an immunohistochemical (IHC) study in prostate cancer found an association between the amount of nuclear LIMK1, higher Gleason scores, and incidence of metastasis [19], suggesting that nuclear LIMK1 may contribute to progression of human cancer. In this study, we sought to determine whether cytoplasmic and/or nuclear LIMK1 localization has a pro-tumorigenic activity in breast cancer cells. Thus, we first performed IHC analysis of LIMK1 expression in normal and malignant human breast specimens and found that LIMK1 expression is increased in both subcellular compartments in human breast cancer, with nuclear levels being highest in those tumors displaying strong cytoplasmic staining. Having demonstrated that LIMK1 can be expressed both cytoplasmically and nuclearly in human breast cancers, we next developed a model system to segregate GFP-tagged LIMK1 to the cytoplasm or the nucleus in MDA-MB-231 breast cancer cells. Using this model, we found that both cytoplasmically-targeted NES-GFP-LIMK1 and nuclearly-targeted NLS-GFP-LIMK1 increased phosphorylation of cofilin, FAK, paxillin, AKT and Erk1/2, both increased cellular invasion, and both enhanced xenograft tumor growth in nude mice. In sum, these studies reveal that LIMK1 has important cytoplasmic and nuclear functions that contribute to breast cancer progression.

## Methods

### Cell lines and cell culture

MDA-MB-231 cells, originally obtained from ATCC, were a kind gift from Dr. Rytis Prekeris, University of Colorado. MDA-MB-231 cells were cultured in high glucose (4.5 g/l), DMEM medium (Invitrogen #11965) supplemented with 15% horse serum (Invitrogen #16050-122), 2.5% fetal bovine serum (FBS, Invitrogen #16000-044) and non-essential amino-acids (Invitrogen #11140-050). Cells were passaged with trypsin three times a week.

### Plasmid constructs

The *Bgl II -Sac II *LIMK1 cDNA fragment (without the start AUG codon) was excised from FPC-1-myc LIMK1 (kind gift from Dr. K Mizuno, Tohoku University, Japan) and ligated into *Bgl II -Sac II *-cut pEGFP-C1 plasmid DNA (Invitrogen Inc., Carlsbad, CA), in-frame and downstream of EGFP, to produce pEGFP-C1-LIMK1. Generation of NES- and NLS-tagged GFP-LIMK1 in the pQCXIN retroviral vector (Clonetech, Inc) was achieved by using oligonucleotides encompassing a Kozak recognition sequence, an AUG start codon (underlined below) and either two NLS or NES sequences, and fusing these oligonucleotides in-frame to amino-terminus of EGFP-C1-LIMK1 template by PCR.

Primers used to generate NLS-GFP-LIMK1:

NLS: 5'-ATTAACCGGTACCATGGCGCCAAAGAAGAAGAGAAAAGTGAGCGGCG GCAGCCCAAAGAAGAAGAGAAAAGTGGTGAGCAAGGGCGAG

LIMK1 Reverse: 5'- TATATTAATTAATGATCAGTTATCTAGATCCG

Primers used to generate NES-GFP-LIMK1:

NES: 5'-ATTAACCGGTACCATGGCGTTAGCACTTAAATTAGCTGGTTTGGACATAG GCGGCTTAGCACTTAAATTAGCTGGTTTGGACATAGTGAGCAAGGGCGAG

LIMK1 reverse: 5'- TATATTAATTAATGATCAGTTATCTAGATCCG

The PCR products and GFP-LIMK1 fragment were then ligated in the *Age I -Pac I *-cut pQCXIN plasmid. The coding sequence for all NLS-GFP-LIMK1, NES-GFP-LMIK1 and GFP-LIMK1 constructs was verified by dideoxy sequencing in the UC Denver Cancer Center DNA Sequencing Core facility.

### Transduction and generation of stable cell pools

Phoenix™ cells (a kind gift from Dr. Heide Ford, UC Denver) were used to package pQCXIN-based retroviruses. For retrovirus production, packaging cells were cultured on 10 cm gelatin-coated plates in 10 ml of DMEM medium supplemented with 10% FBS. Transfection with Effectene (Qiagen #301425) was conducted with 10 μg of retroviral DNA in 80 μl of Enhancer reagent plus 150 μl of Effectene reagent, following the manufacturer's protocol. Virus-containing supernatant was collected at 48 h and 72 h time points, filtered through 0.45 μm syringe filter, aliquoted and stored at -80°C. To infect MDA-MB-231 cells, virus-containing supernatant was diluted in growth medium 1:3, supplemented with polybrene (8 ug/ml) (Sigma cat#107689), and incubated on the target cells. After overnight incubation with the viral supernatant, medium was changed to fresh culture medium. Pools of MDA-MB-231 cells stably-expressing GFP-LIMK1 fusions were selected with G-418 (Invitrogen cat#11811-023). Expression of EGPF from the EGFP-tagged LIMK1 was detected by fluorescence microscopy 48 h-72 h post-infection.

### IHC analysis

For IHC analysis, antigen retrieval was performed by soaking slides in sodium citrate (10 mM solution in phosphate-buffered saline containing 0.1% Tween-20 (PBST), pH 6.0) (Fisher #S279-3) and heating to 120°C for 5 minutes in a decloaker (Biocare Medical). Endogenous peroxidases were blocked by placing slides in 0.3% hydrogen peroxide (Fisherbrand) for 0.5 h, and washed in deionized water. Slides were then washed with PBST (0.1%Tween) and blocked with 10% goat serum for 1 hour. All IHCs were performed using a goat polyclonal antibody that specifically recognizes the C-terminus of LIMK1 [20,21] Primary anti-LIMK1 goat antibody (Santa Cruz #sc8387) diluted 1:100 in the blocking buffer was incubated on samples at 4°C overnight. The slides were then washed in blocking buffer and incubated for 1 h with biotinylated anti-goat IgG secondary antibodies (Jackson Immunoresearch) diluted 1:200 in blocking buffer. All IHC slides were incubated with avidin-biotinylated-horse radish peroxidase (HRP) complexes (Vectastain ABC kit, Vector Laboratories) for 0.5 h, according to the manufacturer's protocol. The antigen-antibody complex was then visualized by a 5-minute treatment with the DAB Plus peroxidase substrate (3,3' diaminobenzidine, Dako Cytomation, # K3468), according to the manufacturer's instructions. Nuclei were visualized with Mayer's hematoxylin (Fisher, 1:10 dilution in water, 30 sec). For mounting, the sections were rinsed in water, dehydrated in graded ethanol (90% ethanol, 3 × 30 sec, 100% ethanol 3 × 30 sec), cleared in xylene (2 × 30 sec), and sealed using Permount (Fisher #SP15-100).

### Western Blots

MDA-MB-231 cell pools stably expressing GFP-LIMK1 fusions were grown in high glucose, DMEM medium supplemented with 15% horse serum, 2.5% fetal bovine serum and non-essential amino-acids, except for lysates prepared for phospho-Erk1/2 and phospho-AKT analysis, which were prepared from cells serum-starved for 24 hours prior to cell harvesting. Cells were washed with cold PBS and lysed on ice in either CHAPS lysis buffer, extraction buffer (EB), or Laemmli Sample buffer (Bio-Rad #161-0737). CHAPs lysis buffer consists of 10 mM CHAPS (Sigma #C926), 50 mM Tris (pH 8.0), 150 mM NaCL, and 2 mM EDTA with 10 μM sodium orthovanadate (Sigma #S6508). EB lysis buffer consists of 10 mM Tris (pH 7.4), 5 mM EDTA, 50 mM NaCl, 1%Triton X100 (Sigma #T9284), and 1 mM DL-Dithiothreitol (DTT) (Sigma #D9779). For lysis with separation of nuclear and cytoplasmic components, 1 × 10^6 ^cells of each cell type were lysed with Pierce NE-PER Nuclear and Cytoplasmic Extraction Reagents. Subcellular fractionation was performed per manufacturer's instructions (Pierce #78833). All lysis buffers were supplemented with Complete protease inhibitor cocktail (Roche #12656900) and PhosSTOP phosphatase inhibitor cocktail (Roche #04906845001), per manufacturer's instructions. Protein concentration was determined using the Bio-Rad DC Protein Assay kit (#500-0116). Total protein (25-100 μg) from each lysate was subjected to sodium dodecyl sulfate 10% polyacrylamide gel electrophoresis (SDS-PAGE) and electrophoresed proteins were transferred to Immobilon-P membranes (Millipore Inc., Bedford MA). Membranes were blocked for 1 - 2 hr in non-fat dry milk (Kroger brand), or ECL Advance blocking agent (GE Healthcare #RPN418V) for phospho-specific primary antibodies. Primary antibodies, rat anti-LIMK1 (100 ng/ml; kindly provided by Dr. James Bamburg, Colorado State University), anti-phospho-LIMK1 (Thr508; 1:2000; Cell Signaling #3841), anti-LIMK1 (1:100,000 Sigma #L-2290), anti-PARP (1:1000; BD Pharmingen #556362), anti-tubulin (1:10,000; Calbiochem #CP06), anti-cofilin (1:500; Abcam #ab54532), anti-phospho-cofilin (Ser3; 1:2000; Cell Signaling #3313), anti-GAPDH (1:40,000; Applied Biosystems #AM4300), anti-FAK (1:1000; Cell Signaling #3285), anti-phospho-FAK (1:4000; Invitrogen #44-626G), anti-paxillin (1:1000; Cell Signaling #2542), anti-phospho-paxillin (Tyr118; 1:1000 Cell Signaling #2541), anti-Src (1:5000; Cell Signaling #2109), anti-phospho-Src (Tyr416; 1:1000; Cell Signaling #2101), anti-Erk1/2 (1:1000; Upstate #06-182), anti-phospho-Erk1/2 (1:1000; Cell Signaling 9101), anti-AKT (1:1000; Cell Signaling #9272), and anti-phospho-AKT (Ser473; Cell Signaling #9271) were incubated overnight at 4°C. Polyclonal goat HRP-conjugated secondary antibodies against mouse, rabbit or rat (Bio-Rad Inc., Hercules, CA, 1:5000 dilution), were incubated on membranes for 1 hr at room temperature. After primary analysis, each blot was stripped using the Chemicon strong reblot reagent (Chemicon, Inc., Temecula, CA) prior to re-probing with additional primary antibodies.

### Immunofluorescence microscopy

MDA-MB-231 cells expressing GFP or the various GFP-LIMK1 fusions were fixed in 4% PFA and then permeabilized with 0.5% Triton X-100 in PBS for 5 min, followed by washing with 100 mM glycine solution three times, 5 min per wash. Cells were then blocked for 1 h - 2 h in PBST with 5% goat serum. Following the blocking incubation, sections were incubated overnight at 4°C with phospho-FAK antibody (Tyr861; Invitrogen #44-626G) diluted 1:100 in blocking buffer. After three 5-minute washes in PBST, sections were incubated for 1 h with a goat IgG secondary antibody (Jackson Immunoresearch) in PBST, followed again by three washes and 1 h counterstain with Alexa Fluor 647-conjugated phalloidin (Invitrogen, #A22287, 1:2000) to visualize the filamentous actin. Slides were sealed using Fluoromount-G (Southern Biotech #0100-01) medium. The slides were imaged via fluorescence microscopy at 40 × magnification (OLYMPUS IX81 inverted microscope at the University of Colorado Denver Light Microscopy Facility utilizing the Intelligent Imaging Slidebook v.4.067 software).

### Invasion assay

Matrigel-based trans-well invasion assays (BD Biosciences cat# 354483) were performed following the manufacturer's guidelines. Briefly, 5 × 10^4 ^MDA-MB-231 cells in DMEM+0.1% BSA were plated in 24-well plates with DMEM+5% FBS as chemo-attractant. After 24 hours, the cells were fixed in 4% PFA and the invading cells on the underside of the filter were stained with Hoechst stain. Invading cells on the bottom of the filters were imaged by fluorescence microscopy. Five high-power fields were counted per filter to score for invasion. Cell number was quantitated with Image J software.

### Nude mouse xenograft tumor assay

Xenograft experiments were conducted in 7-8 week old female nude mice, purchased from the NCI or Harlan Laboratories. MDA-MB-231 cells expressing each of the GFP-LIMK1 fusions as stable pools were harvested in PBS/EDTA and re-suspended in Matrigel (BD Biosciences #356230) at a density of 40 × 10^6 ^cells/ml. For each injection, 2 × 10^6 ^MDA-MB-231 cells were injected bilaterally onto mammary fat pads #5 in a 50 μl volume of Matrigel, with 6-10 animals injected per cell line, per study. Tumor size was assessed by measurements with an electronic caliper. Volume was calculated as 0.52 × length × width × 2. Nude mouse xenograft experiments were performed under animal protocol (#63801707(03)1E), approved by the Animal Care and Use Committee of the University of Colorado Denver.

### Statistical Methods

Statistical analysis of Matrigel invasion assay data was performed in consultation with the Colorado Biostatistics Consortium. The data analysis was generated using SAS software, Version 9.2 (SAS Institute, NC). Briefly, within each experiment, cell count data were standardized by dividing the mean cell count by the control group (GFP). The standardized data was fit to a general linear mixed model. Parameter estimates and statistical test results were obtained using the maximum likelihood method and containment degrees of freedom in SAS 9.2 proc mixed. A global F test for the group effect determined whether any differences existed between group means. Pair-wise comparisons of group means were conducted using the Tukey-Kramer method for multiple comparisons.

Statistical analysis of mouse tumor data sets was performed in consultation with the Biostatistics and Informatics division of the Colorado School of Public Health. All data analyses were performed using SAS software, Version 9.2 (SAS Institute, NC). Briefly, tumor volumes from the entire course of each experiment were used to generate linear mixed regression models. These models were used to analyze the associations between log-tumor volume and cell line-type, over the course of each assay. Pair-wise comparisons of group means were conducted using the Tukey-Kramer method for multiple comparisons. Differences of tumor weights were compared using a one-way ANOVA. A pair-wise comparison was done via a two-sample t-test, and the p-values were calculated based on the specific mean differences and the overall between-animal standard deviation.

## Results

### LIMK1 expression is increased in human breast cancer specimens, with tumors displaying high cytoplasmic LIMK1 levels showing enhanced nuclear LIMK1 staining

**Figure 1:**
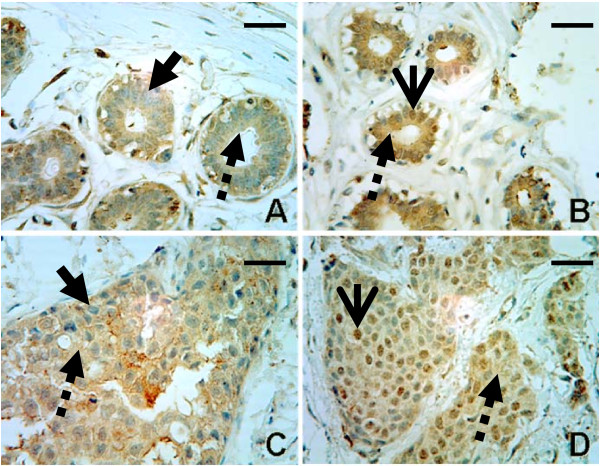
**LIMK1 is detected in the cytoplasm and the nucleus of both normal mammary tissue and human breast cancer tissue**. A total of 67 normal breast tissue samples and 84 breast cancer samples were analyzed for stain localization and intensity. IHCs were performed using goat polyclonal antibody that specifically recognizes the C-terminus of LIMK1. (A) IHC analysis detects mostly faint cytoplasmic LIMK1 staining in normal human mammary tissue (broken arrow). Nuclei are blue with hematoxylin counterstain, without significant LIMK1 stain (solid arrow). Scale bar represents 100 microns. (B) IHC analysis detects cytoplasmic (broken arrow) and nuclear (open arrow) LIMK1 staining in normal human mammary tissue. Nuclei are stained positive with significant LIMK1 stain (open arrow). Scale bar represents 100 microns. (C) IHC analysis detects mostly cytoplasmic LIMK1 staining (broken arrow) in human breast cancer tissue. Nuclei are blue with hematoxylin counterstain, without significant LIMK1 stain (solid arrow). Scale bar represents 100 microns. (D) IHC analysis detects cytoplasmic (broken arrow) and nuclear (open arrow) LIMK1 staining in human breast cancer tissue. Nuclei are stained positive with significant LIMK1 stain (open arrow). Scale bar represents 100 microns.

In order to assess the subcellular compartmentalization of LIMK1 in normal human mammary epithelial cells, as well as human breast cancers, we screened a total of 67 normal breast tissue samples and 84 breast cancer samples for LIMK1 by IHC in several commercially available tissue micro-arrays (TMAs). All normal breast tissue samples that we examined stained positive for LIMK1. The LIMK1 stain within normal breast tissues was found both within the cytoplasm and nucleus of the mammary epithelial cells. In some normal breast tissue samples, the staining appeared to be predominantly cytoplasmic, with limited number of intermingled nuclear-stained cells (Figure 1A). Other samples had both nuclear and cytoplasmic stain (Figure 1B). Similarly, the breast cancer tissue samples were also found to stain predominantly within the cytoplasm (Figure 1C), or within the nucleus and cytoplasm (Figure 1D). Again, as in the normal tissues, the cancer tissues were generally not homogeneous for a given phenotype of LIMK1 localization; however, the tissues could easily be labeled as predominantly cytoplasmic, or predominantly cytoplasmic plus nuclear in nature. Our analysis revealed that 76% of breast cancer tissues could be characterized as strongly stained in the cytoplasm, while only 48% of normal breast tissue was similarly stained (Table 1). Within this subset of strongly cytoplasmic stained tissues, 52% of the strongly cytoplasmic stained breast cancer tissues also expressed nuclear LIMK1, whereas only 27% of the corresponding strong cytoplasmic stained normal breast tissue also expressed nuclear LIMK1 (Table 1).

**Table 1:** IHC scoring for LIMK1 expression and nuclear localization

LIMK1 staining by IHC	Normal (67 samples)	Breast Cancer (84 samples)
Strong Cytoplasmic*	48%	76%
Nuclear staining in those with strong cytoplasmic LIMK1^+^	27% (13%)	52% (39%)

*Percent of total

^+ ^Percent of subset with strong cytoplasmic staining; (% of total)

### In-frame fusion of strong NLS and NES sequences to GFP-LIMK1 imparts restriction to the nuclear and cytoplasmic compartments, respectively

**Figure 2:**
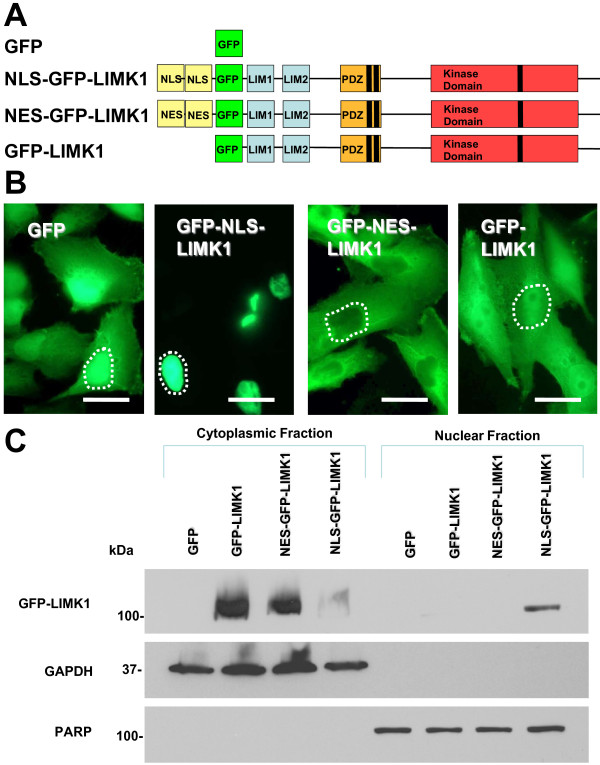
**Subcellular targeting of GFP-LIMK1**. (A) GFP-alone and three GFP-tagged LIMK1 proteins are depicted graphically and color coded. GFP was fused to the N-terminus of the LIMK1 cDNA. Exogenous NLS and NES tags were fused to the N-terminus of GFP. (B) Exogenous NLS and NES sequences target NLS-GFP-LIMK1 and NES-GFP-LIMK1 proteins to the nuclear and cytoplasmic subcellular compartments. Fluorescence microscopy was used to visualize subcellular localization of GFP fluorescence (green) in MDA-MB-231 cells. White-dashed lines are drawn to outline the nucleus of individual cells. Scale bar represents 20 microns. (C) Western blot analysis of cytoplasmic and nuclear fractions from MDA-MB-231 stable transductants. Nuclear segregation is assayed by total PARP. Cytoplasmic segregation is assayed by GAPDH. GFP-tagged LIMK1 is assayed with anti-LIMK1 antibody. Each lane is loaded total nuclear or cytoplasmic extract from 1 × 10^6 ^cells.

Utilizing conventional PCR-based cloning methods, we fused in-frame to the amino-terminus of GFP-LIMK1, either a duplicated NLS motif derived from SV40 Large T-antigen to generate NLS-GFP-LIMK1, or a duplicated NES sequence derived from the inhibitor of cAMP-dependent protein kinase (protein kinase inhibitor [PKI]) to generate NES-GFP-LIMK1 (Figure 2A). Stably-transduced MDA-MB-231 cells were visualized via fluorescence microscopy for GFP localization (Figure 2B). GFP-only expressing cells revlead GFP flurorescence in both cytoplasmic and nuclear compartments (Figure 1B). In contrast, the NLS-GFP-LIMK1 protein was visualized exclusively within the nucleus of the MDA-MB-231 cells (Figure 2B). Conversely, the NES-GFP-LIMK1 protein by GFP visualization was excluded from the nucleus (Figure 2B). Finally, GFP-LIMK1 was expressed in both the cytoplasm and nucleus (Figure 2B). Furthermore, we used biochemical fractionation to corroborate the subcellular localization of these GFP-LIMK1 fusions, and by this approach we found that GFP-LIMK1 and NES-GFP-LIMK1 are detected only in the cytoplasmic fraction, co-purifying with GAPDH, whereas NLS-GFP-LIMK1 was detected predominantly in the nuclear fraction, as defined by poly (ADP-ribose) polymerase (PARP) (Figure 2C). Moreover, the total levels of GFP-LIMK1 and NES-GFP-LIMK1 are each greater than that of NLS-GFP-LIMK1. Thus, utilizing NLS- and NES-tagged GFP-LIMK1, we created a model of predominant nuclear or cytoplasmic GFP-LIMK1 expression.

**Figure 3:**
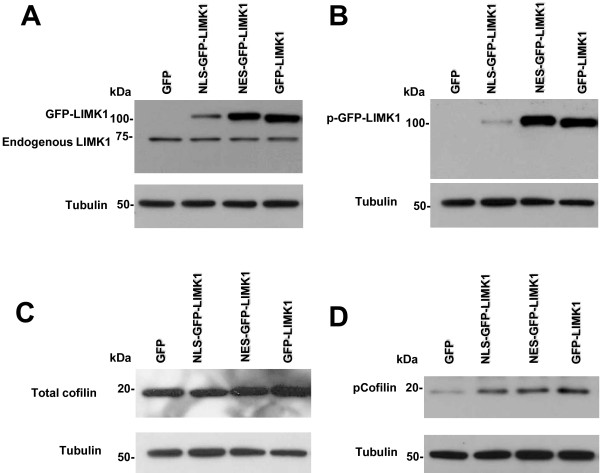
**Expression and phosphorylation status of GFP-LIMK1 fusion proteins and cofilin**. Western blot analysis of whole cell lysates from MDA-MB-231 stable transductants probed for (A) LIMK1 using rat monoclonal antibody and for tubulin using anti-tubulin antibody; (B) probed for phosphoryated LIMK1 at T508 and tubulin (endogenous pLIMK1 is not detectable by Western blot with this antibody); (C) probed for total cofilin and tubulin; and, (D) probed for phosphoryated cofilin at Ser3 and tubulin.

To assess whether equivalent levels of GFP-LIMK1 fusion protein was expressed in these stable pools, we performed Western blot analysis of whole cell lysates from MDA-MB-231 cells stably expressing GFP-only, GFP-LIMK1, NLS-GFP-LIMK1 and NES-GFP-LIMK1 (Figure 3A). These data are consistent with the results shown in Figure 2C, whereby expression of NLS-GFP-LIMK1 is lower compared to expression of GFP-LIMK1 and NES-GFP-LIMK1 (Figure 3A). Comparing exogenous to endogenous LIMK1 expression levels, we find that GFP-LIMK1 and NES-GFP-LIMK1 are expressed about 6-fold higher than endogenous LIMK1, with NLS-GFP-LIMK1 being expressed at approximately the same levels as endogenous LIMK1 (Figure 3A). Since LIMK1 is activated by phosphorylation at T508, we used a phospho-specific antibody against T508 of LIMK1 in Western blot analysis of whole cell lysates to determine the activation status of the various GFP-tagged LIMK1 fusion proteins (Figure 3B). We found T508 phosphorylation levels in GFP-LIMK1 and NES-GFP-LIMK1 to be similar to one another (Figure 3B). However, there is an appreciably smaller level of T508 phosphorylation of the NLS-GFP-LIMK1 protein, relative to others in this group (Figure 3B). The phosphorylation of endogenous LIMK1 was not detectable in this analysis.

### Expression of GFP-LIMK1 proteins increases phosphorylation of cofilin in stable pools of MDA-MB-231 cells

We next tested the ability of the various GFP-tagged LIMK1 fusions to phosphorylate coflin, since this is the key known LIMK1 substrate. We performed Western blot analysis of whole cell lysates from MDA-MB-231 cells that stably express GFP, NLS-GFP-LIMK1, NES-GFP-LIMK1 and GFP-LIMK1 using a total cofilin antibody, and an anti-cofilin antibody that specifically recognizes phosphorylation of Ser3. The amount of Ser3 phosphorylated cofilin is increased to similar levels with expression of all GFP-LIMK1, NLS-GFP-LIMK1 and NES-GFP-LIMK1 proteins, compared to GFP-only control (Figure 3D).

### Nuclear and cytoplasmic LIMK1 expression correlates with activating phosphorylation of FAK signaling components

**Figure 4:**
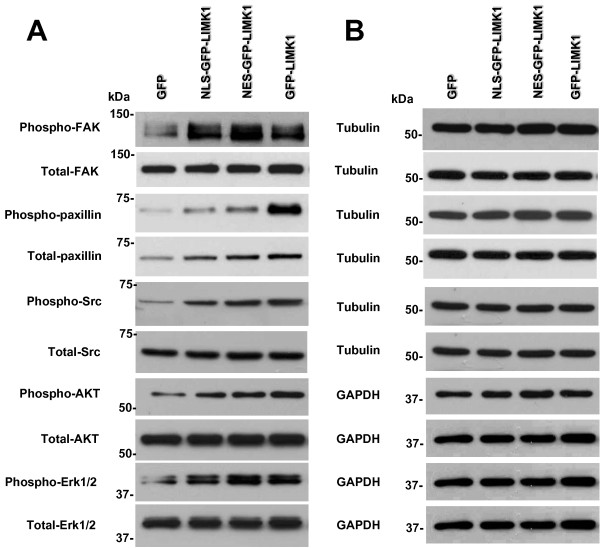
**GFP-LIMK1 fusions increase phosphorylation status of FAK signaling components**. (A) Western blot analysis of whole cell lysates from MDA-MB-231 stable transductants probed with antibodies against pFAK, total FAK, pPaxillin, total Paxillin, pSrc, total Src, pAKT, total AKT, pErk1/2 and total Erk 1/2. (B) Western blot analysis for tubulin or GAPDH for each extract used in panel A, as loading controls.

To determine whether compartment-specific expression of LIMK1 resulted in differential activation of FAK signaling, we performed Western blot analysis of whole cell lysates from MDA-MB-231 cells stably expressing GFP, GFP-LIMK1, NLS-GFP-LIMK1 and NES-GFP-LIMK1, using antibodies that specifically recognize total FAK protein or phosphorylated FAK at Y861 (the site of Src-mediated phosphorylation). The expression levels of total FAK were unchanged with expression of any GFP-tagged LIMK1 protein, compared to GFP-only control (Figure 4A). However, the phosphorylation of FAK was significantly increased with expression of NLS-GFP-LIMK1, NES-GFP-LIMK1, and GFP-LIMK1, compared to GFP-only control (Figure 4A and 4B). Of note, tubulin loading showed that equal amounts of protein were loaded in each lane (Figure 4B).

Similar to FAK total levels, we found no change in expression of total Src, but like FAK we did observe a significant increase in Src phosphorylation at Tyr416 with expression of nuclear and cytoplasmic GFP-tagged LIMK1 proteins (Figure 4A). We next assessed expression of total paxillin and phosphorylation of paxillin at Y118. Expression of all GFP-tagged LIMK1 proteins resulted in increased levels of total paxillin, compared to GFP-only controls (Figure 4A). Phosphorylation of paxillin was also increased, with expression of nuclear and cytoplasmic GFP-tagged LIMK1 proteins, with the greatest increase in cells expressing GFP-LIMK1, compared to GFP-control (Figure 4A).

To assess activation of targets downstream of FAK/paxillin/Src signaling, we next performed Western blots against total Erk1/2 proteins, phosphorylated Erk1/2 at T202/Y204, total AKT, and phosphorylated AKT at Ser473. Similar to FAK and Src, Erk1/2 and AKT total protein levels were unchanged, but phosphorylation of Erk1/2 and AKT was found to be increased with expression of nuclear and cytoplasmic GFP-tagged LIMK1 proteins (Figure 4A and 4B).

**Figure 5:**
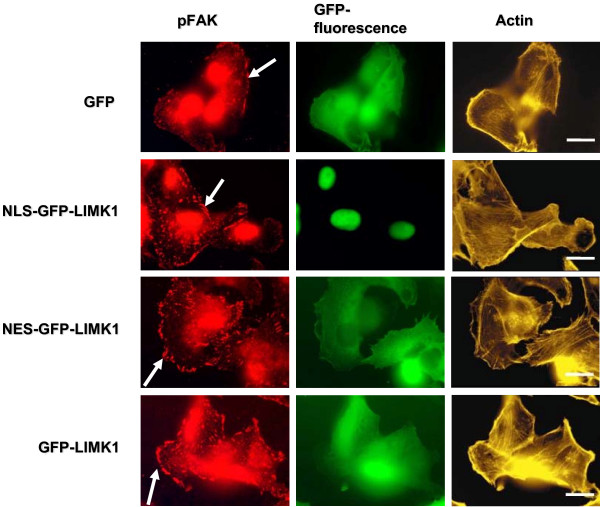
**LIMK1 expression correlates with phosphorylation of FAK in focal adhesions of MDA-MB-231 cells**. MDA-MB-231 cells expressing GFP-only or various GFP-LIMK1 fusions were fixed and stained with antibodies against phospho-FAK (red), and phalloidin against actin (gold). GFP fluorescence is green in this figure. Images were obtained via fluorescence microscopy (non-confocal). Cellular regions with focal adhesion structures are marked by white arrows in the phospho-FAK images. Scale bar represents 20 microns. Note: non-specific binding of secondary antibody stains nuclei red.

Next, we sought to determine whether these distinctly-targeted LIMK1 proteins resulted in differential patterns of activated FAK at focal adhesions structures. To this end, we performed immunofluorescence analysis in MDA-MB-231 cells stably expressing GFP-only, NLS-GFP-LIMK1, NES-GFP-LIMK1 and GFP-LIMK1, using antibodies that specifically recognize phosphorylated FAK at Y861, and fluorescence-conjugated phalloidin to mark actin filaments (Figure 5). Although the secondary antibody alone used in the pFAK study stains the nuclei red nonspecifically (data not shown), we focused on the cytoplasmic pFAK staining pattern. Fluorescence microscopy revealed an increase in both the number of focal adhesions that contain phosphorylated FAK, as well as the relative intensity of staining in the focal adhesions in cells expressing NLS-GFP-LIMK1, NES-GFP-LIMK1, and GFP-LIMK1, compared to GFP-only control (Figure 5).

### Expression of LIMK1 in the nucleus and cytoplasm increases invasion of MDA-MB-231 cells

**Figure 6:**
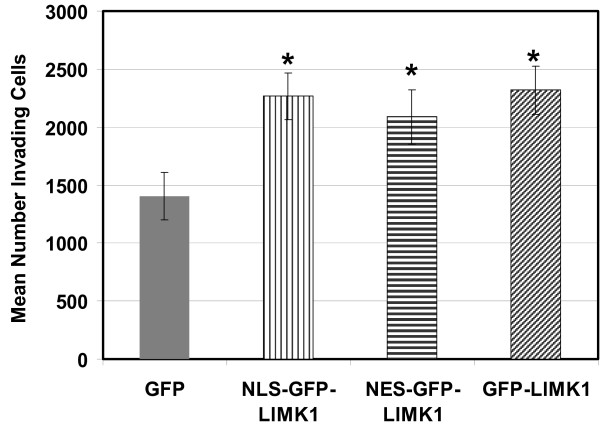
**Cytoplasmic and nuclear LIMK1 enhances invasion**. Invasion assays of MDA-MB-231 cells expressing GFP-only (solid), NLS-GFP-LIMK1 (vertical), NES-GFP-LIMK1 (striped) or GFP-LIMK1 (lines) after 24 hours of invasion through Matrigel-coated invasion chambers. Error bars represent SEM from 8 separate experiments performed in triplicate. Means of each treatment groups differ significantly (p-value less than 0.05) from GFP control (*).

Given that total LIMK1 levels correlate with cellular invasiveness, we next determined whether LIMK1 restricted to distinct subcellular compartments have equal potency in a matrigel-based Boyden chamber cellular invasion assay. The invasion assays of the MDA-MB-231 stable cell lines revealed that expression of GFP-LIMK1 increased invasion ~1.5-fold (mean = 2265 cells; p = 0.002), compared to GFP-only control cells (mean = 1403 cells) (Figure 6). This is consistent with previous studies reporting that ectopic expression of LIMK1 in breast and prostate cancer cell lines increased cellular invasion [13,22]. Moreover, expression of either NLS-GFP-LIMK1 or NES-GFP-LIMK1 also increased cellular invasion to similar levels of ~1.5-fold above GFP-only controls (means equal 2089 and 2319, with p = 0.0008 and p = 0.0075, respectively).

### Nuclear and cytoplasmic LIMK1 enhances xenograft tumor growth of MDA-MB-231 cells

**Figure 7:**
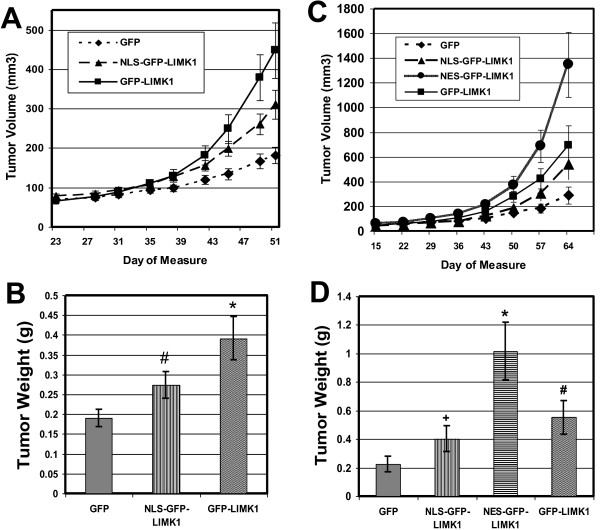
**Expression of nuclear-restricted NLS-GFP-LIMK1, cytoplasmic-restricted NES-GFP-LIMK1, and wild-type GFP-LIMK1 enhance tumor growth**. The results represent two independent experiments (A/B and C/D) in which 2 × 10^6 ^cells MDA-MB-231 cells, in a 50 μl volume of Matrigel, were injected bilaterally onto mammary fat pads #5 of female athymic-nude mice. (A) Tumor volume was estimated by measurements from electronic calipers over the course of the assay, with GFP-only data points shown as diamonds, NLS-GFP-LIMK1 as triangles, and GFP-LIMK1 as squares. Error bars represent SEM, and p-values for all three experimental groups are equal to or less than 0.05, compared to GFP-control. Statistical analysis was performed by mixed model linear regression analysis. (B) Tumor weights were measured upon excision of the tumors in the study shown in (A). The p-value of GFP-LIMK1 is 0.006, compared to GFP-alone (*). The p-value of NLS-GFP-LIMK1, compared to GFP-alone, is 0.37 (#). Statistical analysis of tumor weights was performed by pairwise comparison in one-way ANOVA. (C) Tumor volume was estimated as in (A), with GFP-only data points shown as diamonds, NLS-GFP-LIMK1 as triangles, NES-GFP-LIMK1 as circles and GFP-LIMK1 as squares. Error bars represent SEM, and p-values for GFP-LIMK1 and NES-GFP-LIMK1 are equal to or less than 0.05, compared to GFP-alone. The p-value of NLS-GFP-LIMK1, compared to GFP-alone, is 0.4. Statistical analysis was performed by mixed model linear regression analysis. (D) Tumor weights in (C) were measured upon excision of the tumors. The p-value of NES-GFP-LIMK1 is below 0.0002, compared to GFP-alone (*). The p-values of NLS-GFP-LIMK1 and GFP-LIMK1, compared to GFP-alone, are 0.67 (+) and 0.24 (#), respectively. Statistical analysis of tumor weights was performed by pairwise comparison in one-way ANOVA.

The results of our *in vitro *studies revealed that both cytoplasmic and nuclear localized LIMK1 can impact the aggressiveness of human breast cancer cells. Thus, we hypothesized that the tumor biology would also be impacted by both cytoplasmic and nuclear LIMK1. Our first tumor xenograft study in nude mice specifically tested the contribution of nuclear-plus-cytoplasmic GFP-LIMK1 and nuclear-only NLS-GFP-LIMK1 expression in MDA-MB-231 cells to tumor growth. GFP-only expressing tumors grew to a mean volume of 182 mm^3 ^at 51 days post-injection. We found that the expression of nuclear-restricted NLS-GFP-LIMK1 enhanced tumor growth by ~1.6-fold (mean = 311 mm^3^), compared to GFP-only controls (Figure 7A; p = 0.05). The expression of GFP-LIMK1 increased tumor growth by ~2.5 fold (mean = 448 mm^3^), compared to GFP-alone (Figure 7A; p = 0.0007). At study end, the tumors were removed and weighed. The mean tumor weights for each cell type correlate with the final tumor volume measurements, with GFP-only = 0.19 g (SEM +/- 0.02 g), GFP-LIMK1 = 0.39 g (SEM +/- 0.03 g), and NLS-GFP-LIMK1 = 0.27 g (SEM +/- 0.06 g) (Figure 7B). Notably, we found greater variability in our measurements of extracted tumor weights, compared to tumor volume measurements, as the p-values were found to be 0.37 comparing NLS-GFP-LIMK1 to GFP, and 0.006 comparing GFP-LIMK1 to GFP-only. The greater variability noted in the weight measurements are likely due to the variable contribution of central necrosis, extracellular fluid and/or the variable cropping of adjacent nontumor tissue in the various tumors.

We performed a subsequent experiment to confirm our initial observations and to test the contributions of cytoplasmic NES-GFP-LIMK1 to tumor growth. In this study, the expression of NLS-GFP-LIMK1 enhanced tumor growth similarly to the first experiment (mean = 543 mm^3^), with a ~2-fold enhancement above GFP-only (mean = 290 mm^3^) (Figure 7C; p = 0.4). The expression of GFP-LIMK1 again enhanced tumor formation by ~2.5-fold (mean = 694 mm^3^), above GFP-only control (mean = 290 mm^3^) (Figure 7C; p = 0.006). The expression of NES-GFP-LIMK1 enhanced tumor formation by ~4.7-fold above GFP-controls, from 290 mm^3 ^to 1349 mm^3 ^(Figure 7C; p < 0.001). However, the tumor growth of NES-GFP-LIMK1-expressing cells was not statistically significant compared GFP-LIMK1-expressing cells in this study (p = 0.36). Again, in this experiment, the tumor weight measurements correlated with volume measurements, but also presented greater variability. The tumor mean weights were: GFP-only = 0.22 g (SEM +/- 0.06); NLS-GFP-LIMK1 = 0.40 g (SEM +/- 0.09; p-value = 0.67); and, GFP-LIMK1 = 0.55 g (SEM +/- 0.12; p-value = 0.24) (Figure 7D). The average tumor weight of the NES-GFP-LIMK1 tumors was 1.01 g (SEM +/- 0.2; p-value = 0.0002 vs GFP) (Figure 7D).

## Discussion

The recognized role of LIMK1 is to regulate cell motility and invasion by phosphorylating cofilin, and thus stabilizing actin filaments in the cytoplasm. Not surprisingly, LIMK1 studies have until now focused on the role of LIMK1 within the cytoplasm. However, LIMK1 has also been found in the nucleus and the functional role of nuclear LIMK1 remains unknown. We sought to directly interrogate whether LIMK1 localized to the nucleus (NLS-GFP-LIMK1) displayed tumorigenic properties, compared to LIMK1 targeted to the cytoplasm (NES-GFP-LIMK1) or both subcellular compartments (GFP-LIMK1) (Figure 2). Using this model of GFP-LIMK1 targeted to distinct subcellular compartments in MDA-MB-231 breast cancer cells, we found that both nuclear- and cytoplasmic-targeted GFP-LIMK1 enhanced FAK/paxillin/Src/AKT/Erk signaling, increased cellular invasion, and promoted xenograft tumor growth in nude mice. These data are significant because they show for the first time that LIMK1 targeted to the nucleus evinces similar signaling pathway activation and tumor-promoting properties as LIMK1 targeted either to the cytoplasm or to both subcellular compartments. While it is possible that trace amounts of nuclearly-enforced NLS-GFP-LIMK1 is expressed in the cytoplasm and contributes to its tumor promotion effects, we would point out that direct fluorescence imaging fails to show any NLS-GFP-LIMK1 in the cytoplasm. Moreover, the trace amount detected in the cytoplasmic fraction in the biochemical fractionation study (Figure 2C) is more likely due to protein leak from the nucleus during cell lysis and fractionation. Indeed, the GFP-LIMK1 fusion, without any additional NLS fusion, is clearly evident in the nucleus by direct imaging (Figure 2B), yet upon subcellular fractionation, GFP-LIMK1 is only detected in the "cytoplasmic" fraction, and none appears to be detected in the nuclear fraction (Figure 2C). Such discrepancies between imaging and fractionation studies are best explained by nuclear-to-cytoplasmic leak in these fractionation approaches, since the cytoplasmic leak appears to correlate with the overall strength of interactions with chromatin and nuclear matrix components, such as lamins [23]. Nevertheless, these data support the interesting concept that nuclear LIMK1 contributes to the tumor-promoting effects of total cellular LIMK1.

Using our model of LIMK1 targeted to distinct subcellular compartments, we also observed that expression of LIMK1 in the nuclear and/or cytoplasmic compartments resulted in increased phosphorylation of FAK, paxillin, Src, AKT and Erk1/2 (Figure 4). The mechanism by which cytoplasmically-targeted LIMK1 activates the FAK/paxillin/Src/AKT/Erk signaling pathway is likely via LIMK1 phosphorylation of cytoplasmic cofilin, which stabilizes actin fibers, thus permitting mechanotransduction to activate integrin/FAK signaling [24,25]. Our observation that nuclearly-targeted LIMK1 results in activation of the FAK/paxillin/Src/AKT/Erk signaling pathway is novel, and thus the mechanism by which nuclear LIMK1 stimulates this pathway is less clear. However, since cofilin is known to cycle through the nucleus [26,27], we speculate that nuclear NLS-GFP-LIMK1 could directly phosphorylate nuclear-transiting cofilin, resulting in increased total phospho-cofilin levels (Figure 3D). The increased phospho-cofilin would then act in the manner described above, to activate the FAK signaling pathway. Interestingly, the phospho-cofilin levels are similarly increased in the three GFP-LIMK1 fusions (Figure 3D), despite the much lower level of pT508 NLS-GFP-LIMK1 compared to pT508 NES-GFP-LIMK1 and pT508 GFP-LIMK1 (Figure 3B). These data suggest that cells tolerate a maximal level of steady-state phospho-cofilin, which is known to be regulated by slingshot phosphatase [8]. With regards to the correlation of phospho-cofilin and activated components of the FAK/paxillin/Src/AKT/Erk signaling pathway, we found that phospho-FAK, phospho-Src, phospho-AKT and phospho-Erk did correlate with phospho-cofilin levels (Figure 4). However, phospho-paxillin was very strongly induced in the GFP-LIMK1 cells, and total paxillin levels were modestly induced by all three GFP-LIMK1 fusions (Figure 4). The basis for this marked increase in phospho-paxillin selectively in the GFP-LIMK1 cells is unclear. Nevertheless, expression of all three GFP-LIMK1 fusions resulted in increased FAK/paxillin/Src/AKT/Erk signaling, and increased MDA-MB-231 cellular invasion and xenograft tumor growth in nude mice.

While all three GFP-LIMK1 fusions resulted in an increased and equivalent invasive phenotype (Figure 6), which correlated with cofilin, FAK, Src, AKT, and Erk phosphorylation (Figures 3 &4), the tumor growth response of the different GFP-LIMK1 fusions did not strictly correlate with their cofilin-FAK signaling activity (Figure 7). The *in vitro *invasion data are consistent with previously published reports showing that phospho-cofilin is a key factor regulating cell motility and invasion [13,18], since MDA-MB-231 cells expressing each of the three GFP-LIMK1 fusions displayed equivalent phospho-cofilin (Figure 3D) and cellular invasion (Figure 6) levels. In contrast, these equivalent phospho-cofilin levels and FAK/paxillin/Src/AKT/Erk signaling cannot explain the differential tumor growth generated by the MDA-MB-231 cells expressing the various GFP-LIMK1 fusions (Figure 7). Undoubtedly, a key difference between the *in vitro *invasion assays and the *in vivo *tumor formation studies is that the latter involves multiple tissue interactions. We speculate that a threshold is reached in our system of LIMK1-mediated activation of FAK/paxillin/Src/AKT/Erk signaling as it contributes to tumor growth. Thus, there are likely other, yet uncharacterized LIMK1 pathways that are distinctly affected by nuclear or cytoplasmic LIMK1 that contribute to the differential tumor promoting effects observed *in vivo*. Because LIM-domain proteins often function as nuclear scaffolds that can participate in transcription events [28], one possibility is that nuclear LIMK1 may mediate tumor promoting events via a direct contribution to transcription control. Within the cytoplasm, LIMK1 may mediate tumor progression via effects on p57^Kip2 ^or serum response factor (SRF), both of which are thought to be directly regulated by cytoplasmic LIMK1 [29,30], and both of which are known to influence tumor biology [31,32].

## Conclusions

The data presented here reveal that LIMK1 is expressed in both the cytoplasmic and nuclear compartments in human breast cancer specimens, and that LIMK1 in either cytoplasmic or nuclear compartments contributes to mammary epithelial cell tumorigenesis. Increased LIMK1 expression, targeted to any subcellular compartment, is associated with activation of FAK/paxillin/Src/AKT/Erk signaling and increased activated FAK at focal adhesion complexes. This report provides novel insights into our understanding of the role of LIMK1, and its subcellular localization, in mammary cell tumorigenesis.

## Competing interests

The authors declare that they have no competing interests.

## Authors' contributions

BVM designed the experiments and performed IHC assays, immunofluorescence assays, cell invasion assays, Western blot assays, and xenograft tumorigenicity assays. BVM also wrote the manuscript. KK performed Western blot assays. AGH directed the overall design of the study and participated in the preparation of the manuscript. All authors read, assisted in revision, and approved the final manuscript.
